# Endoscopic release of congenital muscular torticollis in children via a sub-platysmal approach: a retrospective study of 44 cases

**DOI:** 10.3389/fped.2026.1833236

**Published:** 2026-06-16

**Authors:** Yanhua Feng, Qiang Ren, Jing Feng, Qingfeng Ji, Jingyan Li

**Affiliations:** 1 Emergency Surgery, Children’s Hospital of Hebei Province, Shijiazhuang, China; 2 Hebei Provincial Clinical Research Center for Child Health and Disease, Shijiazhuang, China; 3 Emergency Department, The Second Hospital of Hebei Medical University, Shijiazhuang, China

**Keywords:** clinical outcomes, congenital muscular torticollis, endoscopy, minimally invasive surgery, musculoskeletal disorders, pediatric orthopaedics, sternocleidomastoid muscle, sub-platysmal approach

## Abstract

**Background:**

Congenital muscular torticollis (CMT) is a common pediatric musculoskeletal condition resulting from unilateral contracture of the sternocleidomastoid muscle. When conservative treatment fails, surgical intervention is required. Traditional open approaches leave visible cervical scars, prompting the need for minimally invasive alternatives. This study evaluates the clinical outcomes of an innovative endoscopic sub-platysmal release technique for CMT in children.

**Methods:**

We retrospectively analyzed pediatric patients (<10 years) with CMT who underwent endoscopic release via a sub-platysmal approach under general anesthesia after failed at least 6 months of conservative therapy. The procedure utilized a 2.7-mm endoscope and radiofrequency ablation with gravity-fed hemostatic irrigation. Outcomes were assessed using the Cheng and Tang scoring system, including neck rotation deficiency, lateral bending deficiency, craniofacial asymmetry, and head tilt. Operative time, blood loss, and complications were recorded. Minimum follow-up was 30 months.

**Results:**

A total of 44 patients (28 males, 16 females; mean age 3.88 ± 2.15 years) operated between January 2019 and December 2023 were analyzed. The cohort had a mean follow-up of 49.3 ± 8.2 months. Mean operative time was 35.2 ± 6.4 min, and mean blood loss was 4.5 ± 1.2 mL. At final follow-up, the median neck rotation deficiency significantly improved from 26.5° (IQR, 23.1°−29.4°) preoperatively to 3.0° (IQR, 2.5°−4.1°) (*P* < 0.001), and lateral bending deficiency improved from 18.2° (IQR, 15.8°−20.5°) to 2.5° (IQR, 2.0°−3.3°) (*P* < 0.001). According to Cheng and Tang scoring, 42 patients (95.4%) achieved excellent or good outcomes, with earlier intervention demonstrating significantly higher clinical scores. No intraoperative or postoperative complications were observed.

**Conclusion:**

This endoscopic sub-platysmal approach is a safe and effective minimally invasive technique for pediatric CMT, offering excellent functional recovery, minimal blood loss, superior cosmetic outcomes, and a remarkable safety profile. It represents a valuable addition to the surgical armamentarium for pediatric musculoskeletal disorders.

## Introduction

Congenital muscular torticollis (CMT) is a complex movement disorder in children attributed to the unilateral contracture and fibrosis of the sternocleidomastoid (SCM) muscle. This condition eventually results in limited neck activity, tilting of the head toward the affected side, chin rotation toward the contralateral side, craniofacial abnormality, and skull asymmetry if left untreated (1, 2). CMT is one of the most common congenital diseases of the musculoskeletal system in newborns and infants, with an estimated incidence ranging from 0.3% to 2.0% (3, 4). Additionally, CMT is frequently associated with other fetal “packaging disorders”, most notably developmental dysplasia of the hip (DDH), necessitating comprehensive physical and sonographic screening during early diagnosis (5, 6).

The precise etiology of CMT remains somewhat controversial. Historically, it was believed to result from birth trauma leading to hematoma and subsequent ischemic contracture. However, recent ultrastructural studies have identified various cellular components such as early differentiated mesenchymal cells, myoblasts, and myofibroblasts within the pathological SCM tissue, suggesting that the disease may stem from intrauterine developmental abnormalities or a localized compartment syndrome rather than solely mechanical trauma (7).

Non-surgical treatment options for CMT are generally the first-line approach for infants and involve traction instruments, stimulation devices with constraint-induced movement therapy, and intensive stretching exercises (3, 8, 9). Physical therapy is highly effective when initiated early, particularly in the first few months of life (10). If these conservative treatment strategies prove ineffective after a sufficient trial period (typically 6–12 months), or if children present late with significant clinical symptoms such as severe head tilt, facial asymmetry, and cervical spine dysmorphism, surgery becomes necessary to achieve SCM muscle release (11–13).

Traditional open surgeries (unipolar/bipolar release or Z-lengthening) offer direct visualization but leave permanent anterior cervical scars, which remain a primary source of parental dissatisfaction (14–17). To address cosmetic and functional concerns, minimally invasive endoscopic techniques have emerged as excellent alternatives since first described in 1998 (18). Various access routes, including transaxillary and retroauricular approaches, have been optimized to hide scars (19–23). However, transaxillary approaches require a long subcutaneous dissection tract, presenting a steep learning curve and risk of extensive tissue trauma. A direct cervical approach utilizing small, strategically placed portals can offer a more balanced access to the SCM.

This study aims to investigate the clinical effectiveness and safety of a direct, minimally invasive endoscopic sub-platysmal approach under general anesthesia for treating CMT in pediatric patients. By establishing a direct and shorter working tract precisely beneath the platysma muscle, we hypothesized that this endoscopic method would yield excellent clinical and functional correction with optimal cosmetic outcomes and a minimal complication rate over a minimum 30-month follow-up period.

## Materials and methods

### Patients

**Figure 1:**
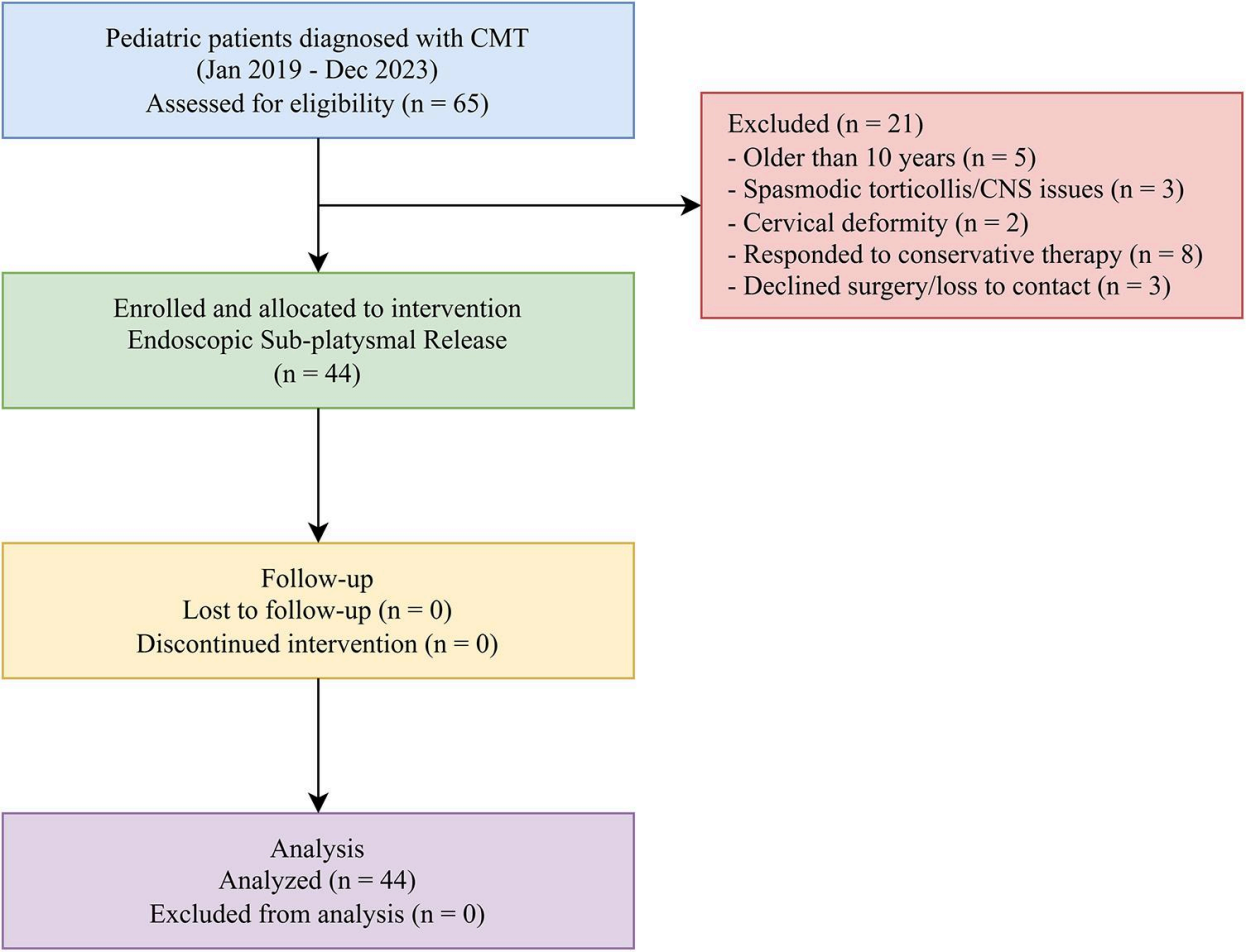
Flowchart detailing the retrospective selection process and follow-up structure of pediatric CMT patients based on STROBE guidelines. The diagram illustrates the initial identification of 65 pediatric patients diagnosed with CMT (January 2019 to December 2023), the subsequent exclusion of 21 patients based on strict clinical criteria (such as age over 10 years, neurological conditions, or successful conservative trials), and the final enrollment, surgical intervention, and robust follow-up of the 44 patients analyzed in this study with zero attrition rate.

This retrospective observational study included pediatric patients with CMT who were admitted to the Department of Orthopedics at Hebei Provincial Children's Hospital between January 2019 and December 2023. Only patients exhibiting defective rotation and limited motion of the neck and head (>15°) who failed conservative treatments involving traction instruments, exogenous stimulation devices, and physical therapy for at least 6 months were eligible. Routine clinical and ultrasonographic screenings were conducted to evaluate for concomitant DDH prior to surgical clearance. The study was reported in accordance with the Strengthening the Reporting of Observational Studies in Epidemiology (STROBE) guidelines (24) (Figure 1).

The exclusion criteria were as follows (1): older than 10 years at the time of surgery (as these older patients frequently exhibit severe secondary skeletal deformities and profound fibrotic contractures requiring extensive open bipolar release or osteotomies rather than endoscopic soft tissue release), (2) acquired torticollis attributed to ocular abnormalities (ruled out via ophthalmology consultation), auditory defects (ruled out via ENT consultation), or central nervous system dysfunction (25), and (3) concomitant cervical deformity such as Klippel–Feil syndrome or atlantoaxial instability, which were strictly excluded using routine preoperative cervical spine radiographs and CT scans. The enrolled participants were accompanied by their guardians, who signed informed consent forms. Additionally, the guardians of the patients completed a preoperative questionnaire with the cooperation of medical staff, including detailed information on the angle of head tilt, degree of rotation, and facial asymmetry. The patients were followed up for at least two and a half years. The study was conducted in accordance with the principles outlined in the Declaration of Helsinki and was approved by the Medical Ethics Committee of the Hebei Provincial Children's Hospital, Shijiazhuang, China.

### Surgical procedure and postoperative management

**Figure 2:**
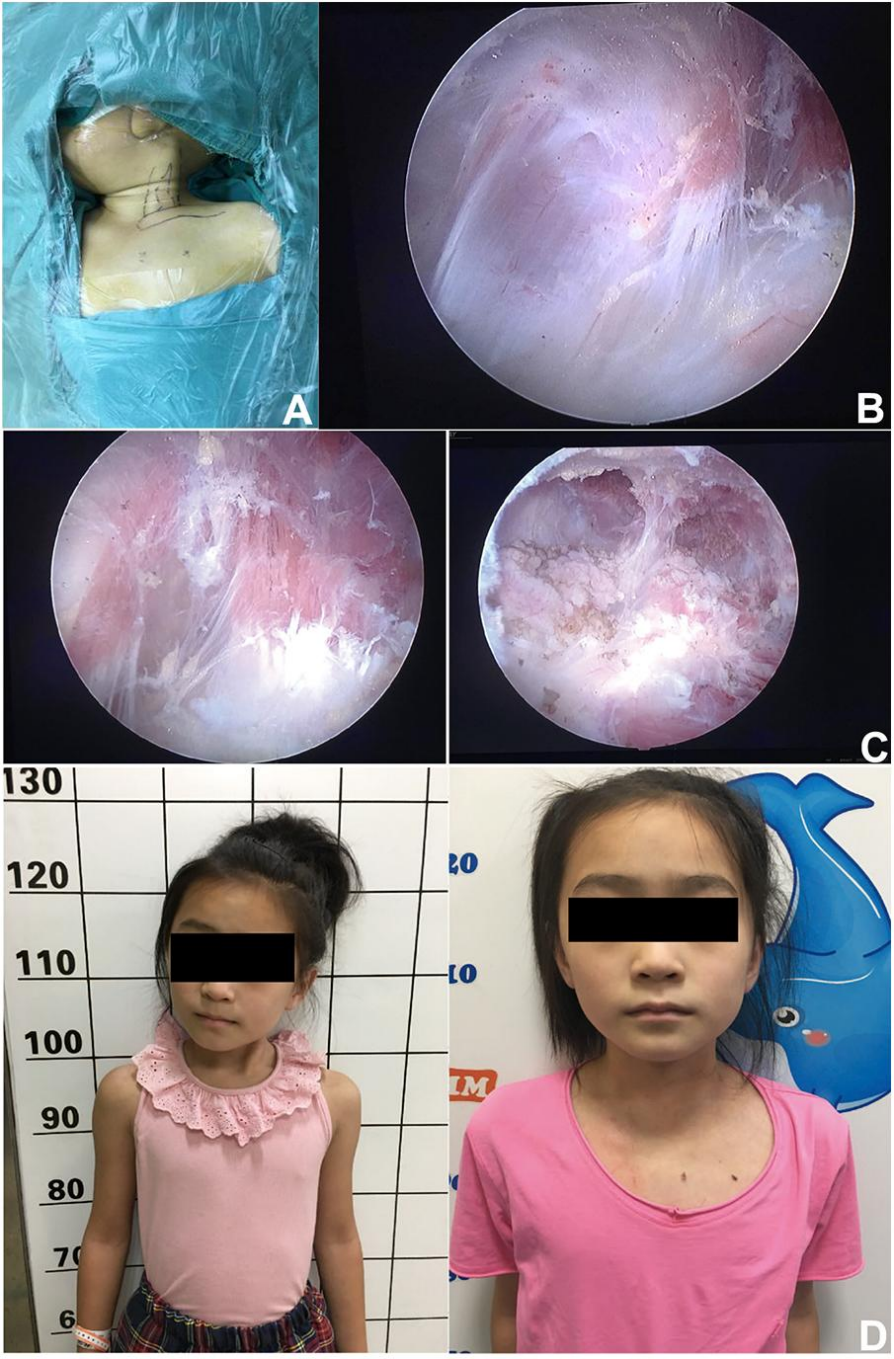
Composite clinical and endoscopic representation of the endoscopic sub-platysmal release for congenital muscular torticollis (CMT). **(A)** Preoperative marking of landmarks and the specific dual-portal approach: the anteromedial portal is located 1–2 cm inferior to the sternoclavicular joint, while the lateral working portal is situated 1–2 cm below the clavicle midpoint. **(B)** Initial endoscopic identification of the tense SCM fibrotic band within the direct sub-platysmal space. **(C)** Endoscopic release demonstrating identification (left panel) and complete transection (right panel) of the clavicular and sternal SCM heads using low-temperature radiofrequency ablation. **(D)** Preoperative facial appearance showing severe head tilt (left panel) and complete structural correction at the 12-month follow-up (right panel).

Patients were placed in a supine position, general anesthesia was induced, and endotracheal intubation was performed to ensure a secured airway. The head was rotated towards the unaffected side with the neck overstretched and the ipsilateral arm abducted to sufficiently expose the surgical region. Surface landmarks such as the sternal notch, clavicle, and sternocleidomastoid muscle were identified and marked. The positions of the two surgical portals were meticulously determined via landmarks and palpation: the anteromedial portal was established 1–2 cm inferior to the sternoclavicular joint, while the anterolateral working portal was positioned 1–2 cm inferior to the mid-clavicular line, accurately matching the body surface markings (Figure 2A).

A 5-mm transverse incision was performed at the marked location, and the approximately 5 cm × 6 cm working region within the sub-platysmal space was initially exposed using a periosteal elevator via blunt dissection. Specifically, a unipolar distal release was systematically performed (22, 26). Crucially, the platysma muscle was preserved and not routinely transected; rather, the working space was bluntly established precisely beneath it to maintain the superficial anatomical architecture. The working region was then flushed with hemostatic liquid comprising 1 mg adrenaline in 3,000 mL saline to maintain a bloodless operative field. Crucially, to prevent fluid extravasation and airway compromise, the fluid was delivered via gravity flow at a controlled height (approximately 60 cm H_2_O) rather than using a high-pressure mechanical pump. The sub-platysmal space was further cleared of loose connective fibers using a 2.7-mm endoscope and a radiofrequency (RF) probe (300-027-100, Stryker Corporation, Kalamazoo, MI, USA) (Figure 2B).

Under conditions of hyperextension, head rotation, and muscular tension, a transverse incision was made after careful identification of the clavicular and sternal heads of the sternocleidomastoid (Figure 2C, left). Strict adherence to the sub-platysmal plane was maintained to protect the external jugular vein superficially and the greater auricular nerve superiorly. Notably, shallower resection was more appropriate than deep resection to avoid damage to the underlying carotid sheath containing major nerves and blood vessels. During endoscopic release, all bleeding was stopped using radiofrequency thermocoagulation to prevent postoperative hemorrhage and hematoma formation. The exact degree of release was determined by the anesthetist by lateral bending and rotation of the neck towards the contralateral side, ensuring complete release of the clavicular and sternal heads of the sternocleidomastoid muscle (Figure 2C, right). During the operation, the blood pressure, heart rate, and oxyhemoglobin saturation of the patients were regularly monitored.

After surgery, patients were immobilized in a custom-made thermoplastic cervical collar maintained in an over-corrected position (5°–10° of contralateral lateral bending and ipsilateral rotation) for 2–4 weeks, depending on the severity of the preoperative contracture. Stretching exercises targeting lateral flexion and rotation towards the affected limits were mandated at a frequency of 3–4 times daily, lasting 10–15 min per session under the guidance of a pediatric physical therapist. Active neck strengthening exercises were initiated sequentially at the third postoperative week to rebuild muscular balance. Postoperative cervical halter traction was not routinely utilized in our cohort to avoid unnecessary discomfort and poor pediatric compliance.

### Outcome assessment

Two independent members of the research group performed preoperative and postoperative physical examinations on all the participants to reduce observer bias. Therapeutic effects were determined using the scoring system developed by Cheng and Tang (27), which assesses the degree of rotational deficit, lateral bending deficit, craniofacial asymmetry, and head tilt. Objective parameters assessed included the degree of rotational deficit and lateral bending deficit measured via a standardized goniometer, while subjective parameters encompassed craniofacial asymmetry and residual head tilt visually appraised. Each aspect received a score of 0, 1, 2, or 3 points based on severity. The total score, which ranges from 0 to 18, is representative of the efficacy of the procedure. A score of 16–18 represents an excellent recovery, 12–15 represents an acceptable (good) outcome, 6–11 indicates a fair result, and <6 indicates a poor outcome. Postoperative incisional cosmetic appearance was independently evaluated using the Vancouver Scar Scale (VSS) at the final follow-up. Additionally, intraoperative parameters including operative time and estimated blood loss were recorded. Complications such as nerve injury, vascular rupture, infection, and skin burns were strictly documented.

### Statistical analysis

All statistical analyses were conducted using SPSS software (version 26.0; IBM Corporation, Armonk, NY, USA). Preoperative and postoperative data were compared. The normality of continuous data distribution was initially assessed using the Shapiro–Wilk test. Normally distributed data were presented as means ± standard deviations (SD), while non-normally distributed data were presented as medians with interquartile ranges (IQR). Preoperative and postoperative rotation deficit and lateral bending deficit data were found to be non-normally distributed and were thus compared using the Wilcoxon signed-rank test. The Kruskal–Wallis test was employed to compare the Cheng and Tang total scores across different age subgroups. A *P* value <0.05 was considered to indicate statistical significance.

## Results

### Participant flow and demographic characteristics

Based on the STROBE screening and enrollment criteria, a total of 44 patients with CMT were included in the final analysis (Figure 1). The demographic profiles of the cohort are outlined in Table 1. The average age at the time of surgery was 3.88 ± 2.15 years (range, 1–9 years). The cohort consisted of 28 (63.6%) males and 16 (36.4%) females, with 24 (54.5%) presenting with right-sided involvement and 20 (45.5%) with left-sided involvement. All patients had failed a minimum of 6 months of prior conservative therapy and were followed up for a mean of 49.3 ± 8.2 months (range, 30–68 months).

**Table 1:** Patient demographics and baseline characteristics.

Characteristic	Value (*n* = 44)
Age at surgery, years (mean ± SD)	3.88 ± 2.15 (range, 1–9)
Gender (male/female), *n* (%)	28 (63.6%)/16 (36.4%)
Affected side (right/left), *n* (%)	24 (54.5%)/20 (45.5%)
Follow-up interval, months (mean ± SD)	49.3 ± 8.2 (range, 30–68)
Failure of prior conservative therapy	44 (100%)

### Intraoperative and perioperative outcomes

The surgical procedures were successfully completed in all patients without the need for conversion to open surgery. As summarized in Table 2, the mean operative time was 35.2 ± 6.4 min and the mean intraoperative blood loss was 4.5 ± 1.2 mL, underlining the highly minimally invasive and tissue-sparing nature of this sub-platysmal endoscopic technique.

**Table 2:** Summary of perioperative parameters and functional outcomes following endoscopic sub-platysmal release.

Parameter	Preoperative median (IQR)	Postoperative median (IQR)	*P*-value
Rotation deficiency (degrees)	26.5° (23.1°–29.4°)	3.0° (2.5°–4.1°)	<0.001
Lateral bending deficiency (degrees)	18.2° (15.8°–20.5°)	2.5° (2.0°–3.3°)	<0.001
Operative time (minutes)	–	35.2 ± 6.4 (mean ± SD)	–
Intraoperative blood loss (mL)	–	4.5 ± 1.2 (mean ± SD)	–
Postoperative VSS score	–	1.0 (0.0–1.0)	
Complication rate (%)	–	0 (0%)	–

### Functional and range of motion recovery

**Figure 3:**
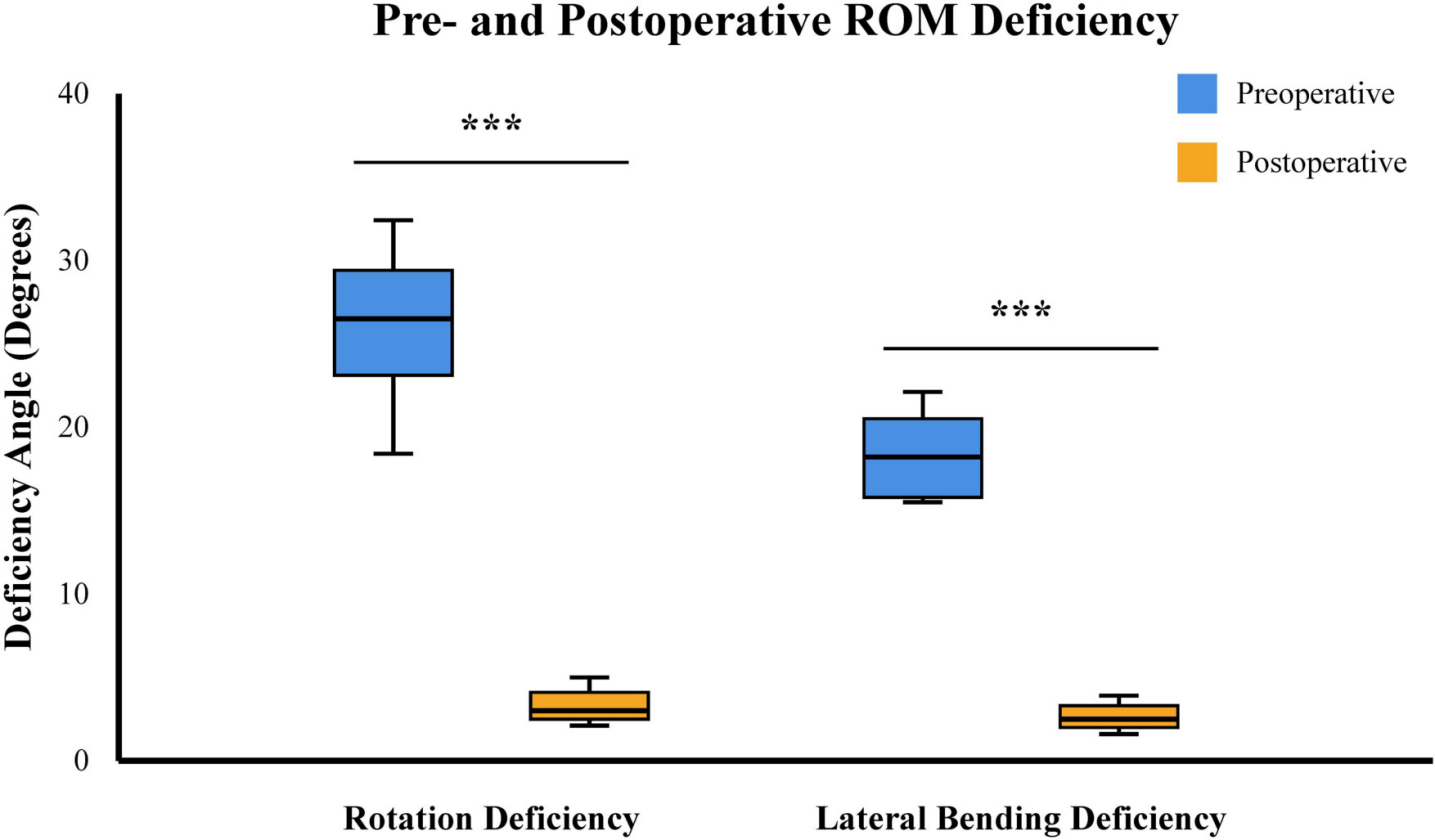
Box-and-whisker plots demonstrating the substantial improvements in neck range of motion (ROM) deficiency before and after endoscopic release (*n* = 44). The plots display the median, interquartile ranges, and ranges for both neck Rotation Deficiency and Lateral Bending Deficiency in degrees. Preoperative and final postoperative follow-up parameters were compared using the Wilcoxon signed-rank test. ***P < 0.001 indicates a highly significant therapeutic improvement.

**Figure 4:**
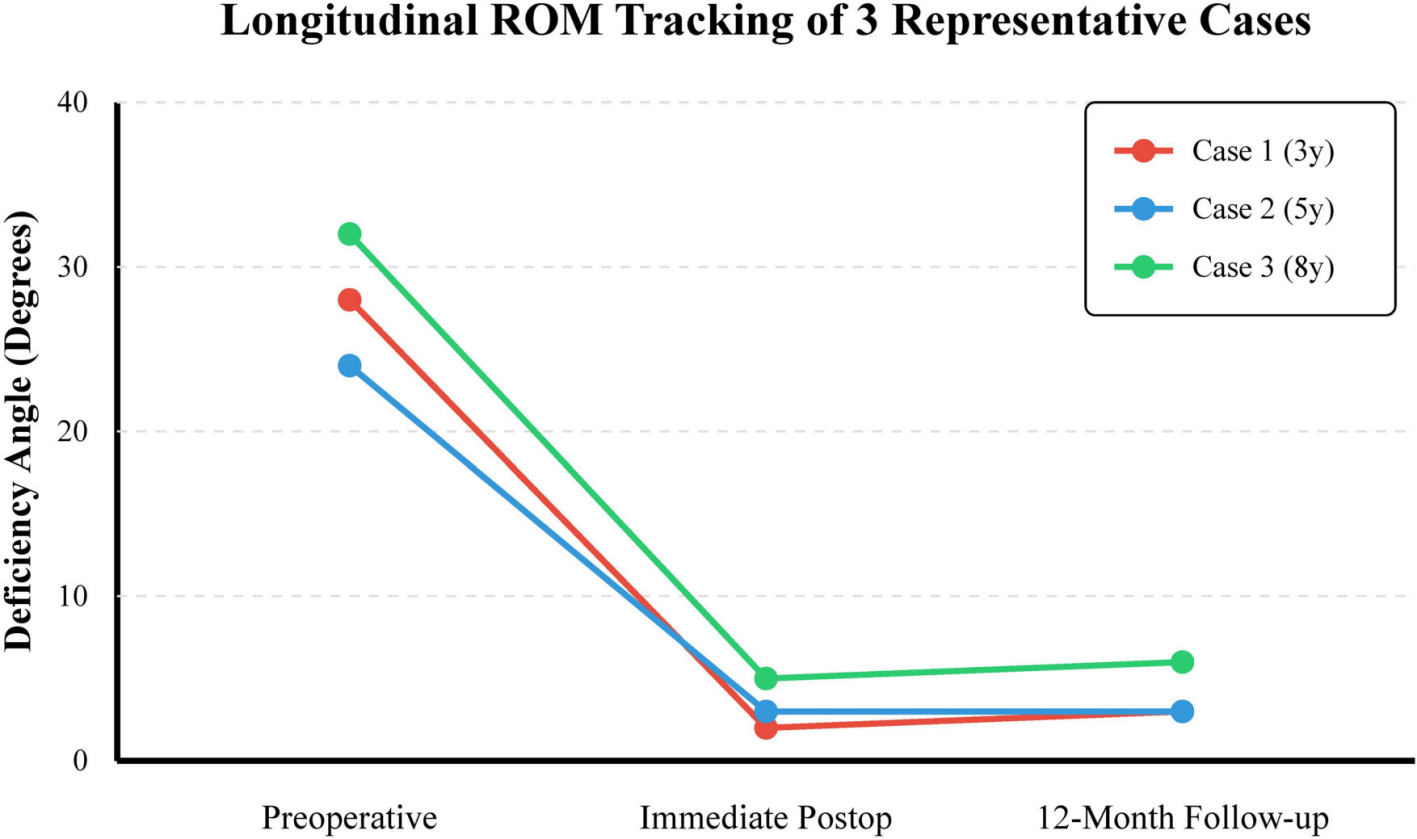
Longitudinal range of motion (ROM) tracking curves of three representative pediatric cases at three distinct evaluation phases. Deficit angles (degrees) are plotted preoperatively, immediately postoperatively, and at the 12-month follow-up for Case 1 (3-year-old toddler), Case 2 (5-year-old preschooler), and Case 3 (8-year-old school-age child). The curves demonstrate rapid immediate post-surgical gains that remain stable and secure over long-term physical rehabilitation.

At the final follow-up, no residual band or head tilt was observed in the majority of the children (Figure 2D). As detailed in Table 2 and Figure 3, the median of neck rotation deficiency had significantly decreased from 26.5° (IQR, 23.1°−29.4°) preoperatively to 3.0° (IQR, 2.5°−4.1°), accompanied by a decreased limitation of lateral bending from 18.2° (IQR, 15.8°−20.5°) preoperatively to 2.5° (IQR, 2.0°−3.3°) (*P* < 0.001). The robust longitudinal functional recovery of Range of Motion (ROM) over a 12-month period for three representative patients is explicitly demonstrated in Table 3 and Figure 4, showing substantial immediate postoperative gains that were effectively maintained throughout rehabilitation.

**Table 3:** Longitudinal follow-up of range of motion in three representative cases.

Patient (age)	Parameter (degrees)	Preoperative	Immediate postoperative	12-Month follow-up
Case 1 (3 years)	Rotation deficiency	28	2	3
	Lateral bending deficiency	20	1	2
Case 2 (5 years)	Rotation deficiency	24	3	3
	Lateral bending deficiency	17	2	2
Case 3 (8 years)	Rotation deficiency	32	5	6
	Lateral bending deficiency	22	4	4

### Clinical success and age subgroup analysis

**Figure 5:**
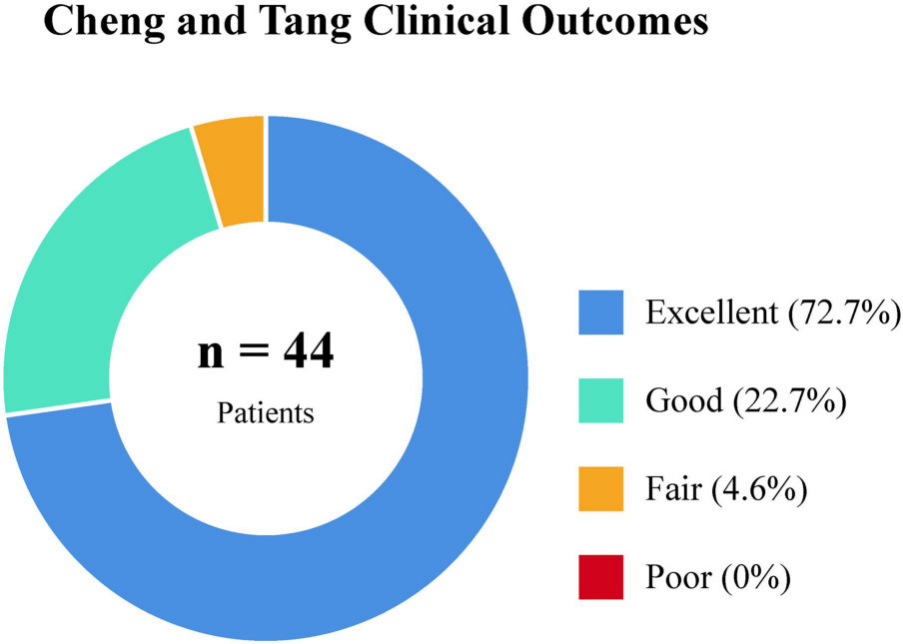
Doughnut chart illustrating the distribution of overall clinical success according to the Cheng and Tang scoring system (*n* = 44). Excellent outcomes (scores of 16–18) were achieved in 72.7% of patients (*n* = 32), and good outcomes (scores of 12–15) were observed in 22.7% (*n* = 10), reflecting a total clinical success rate of 95.4%. Only 2 patients (4.6%) presented with fair outcomes, and none experienced poor outcomes.

**Figure 6:**
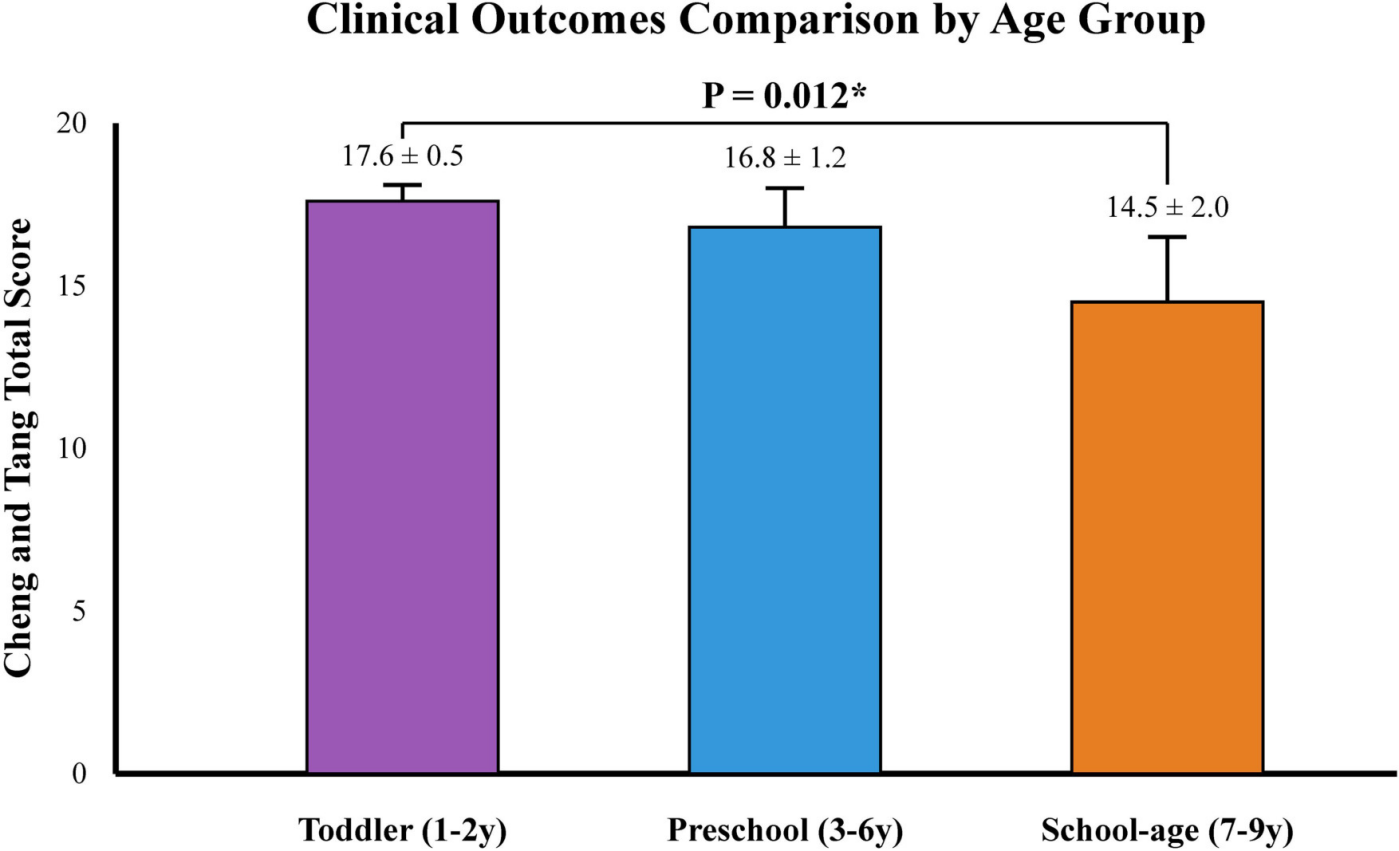
Impact of patient age at the time of surgery on the final postoperative Cheng and Tang clinical total scores. Bar charts display the mean ± standard deviation scores across three distinct age groups: Toddlers (1–2 years, *n* = 14), Preschool-aged children (3–6 years, *n* = 22), and School-aged children (7–9 years, *n* = 8). Statistical comparison using the Kruskal–Wallis test (*P = 0.012) demonstrates that younger children achieve significantly superior therapeutic and functional outcomes, reinforcing the critical importance of early surgical intervention.

Assessment of patients using the scoring system developed by Cheng and Tang (27) revealed that 42 patients (95.4%) had excellent or good outcomes and 2 patients (4.56%) had a fair outcome; no patients had poor outcomes (Figure 5). The two patients demonstrating a “fair” outcome were aged 8 and 9 years at the time of surgery, both presenting with severe preoperative deficits (rotation deficiency > 30°) and a prolonged disease history. Subgroup analysis regarding the impact of surgical age on functional outcomes revealed significant differences. Patients categorized into the Toddler group (1–2 years) exhibited the highest Cheng and Tang total scores compared to the Preschool (3–6 years) and School-age (7–9 years) groups (*P* = 0.012), underscoring the clinical advantage of earlier surgical intervention (Table 4, Figure 6).

**Table 4:** Comparison of postoperative Cheng and Tang scores across age groups.

Age Subgroup	N	Cheng and Tang total score (mean ± SD)	*P*-value (Kruskal–Wallis)
Toddler (1–2 years)	14	17.6 ± 0.5	0.012*
Preschool (3–6 years)	22	16.8 ± 1.2	
School-age (7–9 years)	8	14.5 ± 2.0	

### Safety evaluation and aesthetic outcomes

Crucially, the procedure demonstrated an excellent safety profile. No intraoperative or postoperative complications, such as vascular rupture, nerve injury (including greater auricular nerve neuropraxia), hematoma compression, radiofrequency skin burns, symptomatic fluid extravasation, airway compromise, or incisional wound infections, were observed throughout the follow-up period. Moreover, the aesthetic outcomes were highly satisfactory, with a median postoperative Vancouver Scar Scale (VSS) score of 1.0 (IQR: 0.0–1.0) at the final follow-up. This objective finding confirms excellent cosmetic healing without hypertrophic scarring.

## Discussion

CMT is one of the most common congenital diseases of the musculoskeletal system in newborns and infants. Left untreated, the continuous unilateral contracture of the SCM muscle inevitably leads to progressive craniofacial asymmetry, compensatory scoliosis, and significant psychosocial distress for the child. While conservative management, primarily manual stretching and physical therapy, remains the gold standard for infants under 1 year of age (8–13), surgical intervention is unequivocally indicated for children presenting late or those who fail to progress after a trial of conservative therapy. The primary goal of surgery is to achieve complete release of the restricting fibrotic bands while minimizing irogenic morbidity and maximizing cosmetic outcomes.

Historically, open surgical approaches such as unipolar release (mastoid or clavicular), bipolar release, and Z-lengthening of the SCM were the mainstays of treatment (28–31). While these procedures offer direct visualization and thorough release, they are inherently associated with significant drawbacks. Open unipolar or bipolar releases inevitably leave conspicuous transverse or oblique cervical scars, which can be a source of long-term dissatisfaction for patients and parents. Furthermore, bipolar release completely detaches the SCM, sometimes leading to an aesthetically unpleasing “hollow” neck contour or a palpable mass due to muscle retraction (14).

To address these cosmetic and functional concerns, minimally invasive endoscopic techniques have gained substantial traction (18, 32). Various access routes have been explored, most notably the transaxillary (22, 23) and retroauricular approaches (18). While the transaxillary approach excellently conceals the scar within the axillary fold, it requires the creation of a long subcutaneous working tract. This extended dissection increases the risk of bleeding, requires a steep surgical learning curve, and can make controlling the SCM clavicular head challenging due to the tangential working angle (23).

The present study describes a novel and direct endoscopic approach for CMT surgery in children utilizing the sub-platysmal space. This direct cervical approach strikes an optimal balance. By placing a micro-incision (5 mm) low on the clavicular line, the cosmetic result is highly acceptable, often hiding within natural skin creases. Similar direct cervical portal placements have been successfully utilized and validated by Wang et al. (26) in their endoscopic release utilizing radiofrequency for teenagers, highlighting the reproducibility and anatomic safety of this approach. Simultaneously, compared to the transaxillary route, it provides a much shorter, direct, and safer trajectory to the SCM heads. The current study has revealed the advantages of endoscopic repair under this specific tissue plane, which provides an olighemorrhagic field when combined with gravity-fed epinephrine-saline irrigation and precise RF ablation.

When directly comparing our outcomes with contemporary endoscopic series, such as the endoscopic subcutaneous approaches reported by Wang et al. (19) and Li and Xing (20), our cohort exhibited identically high success rates (>95% excellent/good outcomes) but with a markedly shortened operative time (mean 35.2 min) and a zero complication rate. Compared to established traditional open surgeries, our endoscopic sub-platysmal technique significantly minimizes intraoperative blood loss, dramatically reduces the VSS scar score, and completely prevents the postoperative “hollow-neck” deformity often associated with aggressive open bipolar SCM excisions.

Safety is the paramount concern in endoscopic neck surgery. The use of general anesthesia with endotracheal intubation, as performed in our cohort, is absolutely essential for pediatric patients. It ensures complete airway protection against potential fluid extravasation, maintains strict patient immobility, and allows the anesthetist to safely manipulate the head for dynamic intraoperative testing of the release. Furthermore, we emphasize the use of gravity-flow fluid administration rather than pressurized pumps to strictly prevent fluid tracking into the mediastinum or causing airway compression. Advanced electrosurgical technology, specifically low-temperature RF devices, significantly reduced the occurrence of intraoperative thermal injuries to adjacent structures, yielding a 0% complication rate in our series.

A further advantage of the approach described in this study is the remarkable postoperative functional improvement, with good or excellent outcomes observed in 95.4% of patients according to the rigorous Cheng and Tang scoring system. Postoperative rehabilitation exercises led to rapid improvements in neck rotation (improving from a median 26.5°–3.0° deficiency) and craniofacial symmetry. Notably, our age-stratified subgroup analysis clearly demonstrated that earlier intervention (toddlers aged 1–2 years) yielded superior functional and aesthetic scoring. The suboptimal recovery observed in the two “fair” outcome cases (aged 8 and 9) is likely attributable to deeply entrenched secondary fascial contractures and mild irreversible adaptive cervical skeletal changes due to the delayed intervention, reiterating the necessity of timely surgical referrals once conservative measures plateau.

The strengths of this study lie in the detailed statistical analysis of the clinical effects of our surgical approach and the long follow-up period (mean 49.3 months) that maximized the reliability of the results. Continuous monitoring of patients confirmed the durability of the release without recurrence. Nevertheless, the study has several limitations. First, this is a single-center retrospective analysis lacking a direct control group (e.g., an open surgery cohort). Second, while our complication rate was zero, the relatively small sample size (*n* = 44) means rare complications might not have been captured. Future validation through multicenter prospective randomized controlled trials is required to definitively compare this sub-platysmal endoscopic approach with transaxillary and open techniques. Third, due to strict institutional privacy regulations regarding pediatric patients, we were unable to include clinical videos demonstrating the dynamic postoperative cervical range of motion, which limits the visual presentation of our functional outcome.

## Conclusion

The results of the present study suggest that endoscopic release via the direct cervical sub-platysmal space is a highly secure and reliable surgical approach for children with CMT. When performed under general anesthesia with proper fluid management and RF technology, it significantly improves functional range of motion and cosmetic outcomes with minimal surgical trauma.

## Data Availability

The original contributions presented in the study are included in the article/Supplementary Material, further inquiries can be directed to the corresponding author.

## References

[1] Ploeger MM Trillhaase C Rommelspacher C Bornemann R Ossendorf R Placzek R. Surgical treatment of congenital muscular torticollis. Oper Orthop Traumatol. (2023) 35(3–4):188–94. 10.1007/s00064-023-00805-x 37079025 PMC10247838

[2] Moses V Deville C Simpkins S Wang J Marlow T Holley C. Effects of adherence to treatment for repositioning therapy, physical therapy, and cranial remolding orthoses in infants with cranial deformation. J Rehabil Assist Technol Eng. (2024) 11:20556683241250310. 10.1177/20556683241250310 38694843 PMC11062220

[3] Sargent B Coulter C Cannoy J Kaplan SL. Physical therapy management of congenital muscular torticollis: a 2024 evidence-based clinical practice guideline from the American physical therapy association academy of pediatric physical therapy. Pediatr Phys Ther. (2024) 36(4):370–421. 10.1097/pep.0000000000001114 39356257

[4] Sargent B Kaplan SL Coulter C Baker C. Congenital muscular torticollis: bridging the gap between research and clinical practice. Pediatrics. (2019) 144(2). 10.1542/peds.2019-0582 31350358 PMC6855899

[5] Tien YC Su JY Lin GT Lin SY. Ultrasonographic study of the coexistence of muscular torticollis and dysplasia of the hip. J Pediatr Orthop. (2001) 21(3):343–7. 10.1097/01241398-200105000-00016 11371818

[6] Minihane KP Grayhack JJ Simmons TD Seshadri R Wysocki RW Sarwark JF. Developmental dysplasia of the hip in infants with congenital muscular torticollis. Am J Orthop. (2008) 37(9):E155–8; discussion E8. PMID: 18982188. 18982188

[7] Tang S Liu Z Quan X. Relationship between blood supply of sternocleidomastoid muscle and congenital muscular torticollis. Chin J Pediatr Surg. (2001) 22(1):19–20.

[8] Antares JB Jones MA Chak NTN Chi Y Li H Li M. Efficacy of non-surgical, non-pharmacological treatments for congenital muscular torticollis: a systematic review and meta-analysis. BMC Musculoskelet Disord. (2025) 26(1):178. 10.1186/s12891-025-08407-3 39979901 PMC11844190

[9] Desai S Sharath HV. Effect of pediatric physical therapy interventions on congenital muscular torticollis: a systematic review. Cureus. (2024) 16(9):e69572. 10.7759/cureus.69572 39421128 PMC11486520

[10] Rodríguez-Huguet M Rodríguez-Almagro D Rosety-Rodríguez M Vinolo-Gil MJ Ayala-Martínez C Góngora-Rodríguez J. Effectiveness of the treatment of physiotherapy in the congenital muscular torticollis: a systematic review. Children. (2023) 11(1). 10.3390/children11010008 PMC10814226 38275429

[11] Castilla A Gonzalez M Kysh L Sargent B. Informing the physical therapy management of congenital muscular torticollis clinical practice guideline: a systematic review. Pediatr Phys Ther. (2023) 35(2):190–200. 10.1097/pep.0000000000000993 36637442

[12] Li S Zhao W Sun X. Comment on “traditional Chinese medicine (TCM) massage for the treatment of congenital muscular torticollis (CMT) in infants and children: a systematic review and meta-analysis”. Complement Ther Clin Pract. (2022) 46:101537. 10.1016/j.ctcp.2022.101537 35077927

[13] Luther BL. Congenital muscular torticollis. Orthop Nurs. (2002) 21(3):21–7; quiz 7–9. 10.1097/00006416-200205000-00005 12101935

[14] Funao H Isogai N Otomo N Yamanouchi K Mizukoshi R Fujita N. Clinical results after release of sternocleidomastoid muscle surgery for neglected congenital muscular torticollis-unipolar vs. bipolar release surgery. J Clin Med. (2023) 13(1). 10.3390/jcm13010131 38202137 PMC10780082

[15] Wirth CJ Hagena FW Wuelker N Siebert WE. Biterminal tenotomy for the treatment of congenital muscular torticollis. Long-term results. J Bone Joint Surg Am. (1992) 74(3):427–34. 10.2106/00004623-199274030-00015 1548271

[16] Akazawa H Nakatsuka Y Miyake Y Takahashi Y. Congenital muscular torticollis: long-term follow-up of thirty-eight partial resections of the sternocleidomastoid muscle. Arch Orthop Trauma Surg. (1993) 112(5):205–9. 10.1007/bf00451875 8217454

[17] Beek DM van Vlimmeren L Bruggink R Pelsma M Xi T Nienhuijs M. The effect of combined surgery and physiotherapy on the facial asymmetry in patients with congenital muscular torticollis: a retrospective cohort study. Int J Oral Maxillofac Surg. (2024) 53(11):919–24. 10.1016/j.ijom.2024.04.009 38734490

[18] Burstein FD Cohen SR. Endoscopic surgical treatment for congenital muscular torticollis. Plast Reconstr Surg. (1998) 101(1):20–4; discussion 5–6. 10.1097/00006534-199801000-00004 9427912

[19] Wang XW Yao ZM Zhou DM Yang YJ Guo D Zhang L. Clinical efficacy of arthroscopic minimally invasive treatment in children with congenital muscular torticollis: a retrospective study. J Pediatr Surg. (2025) 60(5):162268. 10.1016/j.jpedsurg.2025.162268 40086158

[20] Li W Xing S. Endoscopic minimally invasive treatment of congenital muscular torticollis in children. J Orthop Surg Res. (2024) 19(1):470. 10.1186/s13018-024-04971-x 39123203 PMC11312245

[21] Kim TH Kim YC Choi JW. Unipolar myomectomy for congenital muscular torticollis: a retrospective study. J Craniomaxillofac Surg. (2024) 52(6):763–71. 10.1016/j.jcms.2024.03.034 38616143

[22] Pan P. The transaxillary subcutaneous endoscopic sternocleidomastoid muscle division as an approach for the surgical treatment of congenital muscular torticollis in children. Indian J Otolaryngol Head Neck Surg. (2020) 72(1):123–7. 10.1007/s12070-019-01770-3 32158668 PMC7040101

[23] Dutta S Albanese CT. Transaxillary subcutaneous endoscopic release of the sternocleidomastoid muscle for treatment of persistent torticollis. J Pediatr Surg. (2008) 43(3):447–50. 10.1016/j.jpedsurg.2007.10.008 18358280

[24] Skrivankova VW Richmond RC Woolf BAR Yarmolinsky J Davies NM Swanson SA. Strengthening the reporting of observational studies in epidemiology using Mendelian randomization: the STROBE-MR statement. J Am Med Assoc. (2021) 326(16):1614–21. 10.1001/jama.2021.18236 34698778

[25] Tomczak KK Rosman NP. Torticollis. J Child Neurol. (2013) 28(3):365–78. 10.1177/0883073812469294 23271760

[26] Wang JL Qi W Liu YJ. Endoscopic release of congenital muscular torticollis with radiofrequency in teenagers. J Orthop Surg Res. (2018) 13(1):100. 10.1186/s13018-018-0801-6 29720210 PMC5932792

[27] Cheng JC Wong MW Tang SP Chen TM Shum SL Wong EM. Clinical determinants of the outcome of manual stretching in the treatment of congenital muscular torticollis in infants. A prospective study of eight hundred and twenty-one cases. J Bone Joint Surg Am. (2001) 83(5):679–87. 10.2106/00004623-200105000-00006 11379737

[28] Bajaj J Ratre S Parihar VS Agarwal P Yadav Y Sharma D. Bipolar release of sternocleidomastoid for congenital muscular torticollis. Neurol India. (2023) 71(3):427–30. 10.4103/0028-3886.378659 37322734

[29] Kumar S. Surgical management of congenital muscular torticollis. Neurol India. (2023) 71(3):415. 10.4103/0028-3886.378691 37322730

[30] Choi JM Seol SH Kim JH Chung CM Park MC. Age group-specific improvement of vertebral scoliosis after the surgical release of congenital muscular torticollis. Arch Plast Surg. (2024) 51(1):72–9. 10.1055/a-2168-4606 38425855 PMC10901601

[31] Patwardhan S Shyam AK Sancheti P Arora P Nagda T Naik P. Adult presentation of congenital muscular torticollis: a series of 12 patients treated with a bipolar release of sternocleidomastoid and Z-lengthening. J Bone Joint Surg Br. (2011) 93(6):828–32. 10.1302/0301-620x.93b6.26232 21586785

[32] Lee IJ Lim SY Song HS Park MC. Complete tight fibrous band release and resection in congenital muscular torticollis. J Plast Reconstr Aesthet Surg. (2010) 63(6):947–53. 10.1016/j.bjps.2009.05.017 19539550

