# Supplementary figures and images for: Endoscopic release of congenital muscular torticollis in children via a sub-platysmal approach: a retrospective study of 44 cases
Source: Front Pediatr. 2026 Jun 16;14:1833236. doi: 10.3389/fped.2026.1833236 (PMC13315177; doi:10.3389/fped.2026.1833236)

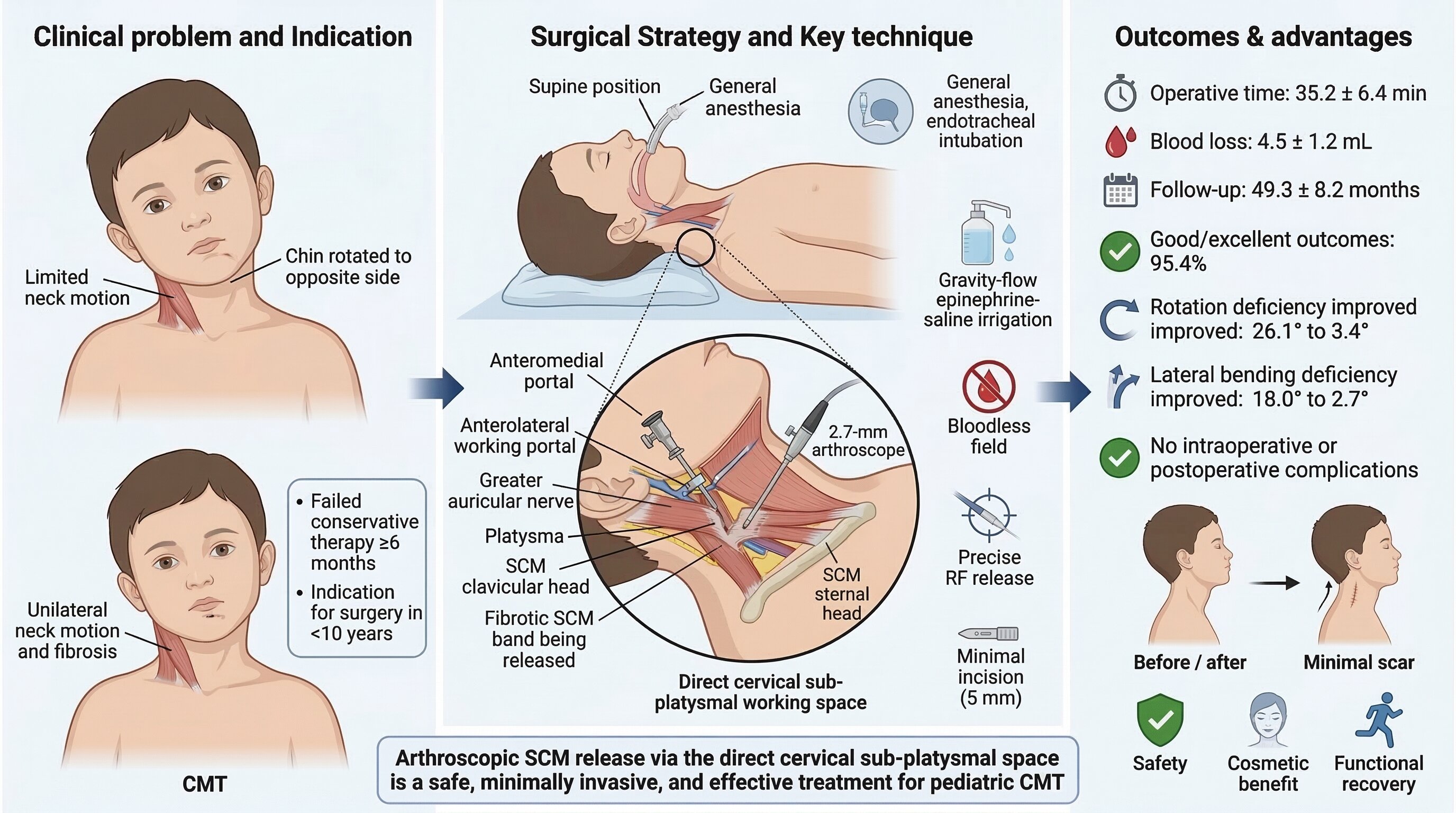

Supplement: Supplementary file 1 [file Image1.jpeg]
