# Supplementary material for: Endoscopic release of congenital muscular torticollis in children via a sub-platysmal approach: a retrospective study of 44 cases
Source: Front Pediatr. 2026 Jun 16;14:1833236. doi: 10.3389/fped.2026.1833236 (PMC13315177; doi:10.3389/fped.2026.1833236)
Supplement: Supplementary file 2 [file Table1.docx]

**Table 1. Patient Demographics and Baseline Characteristics**

| **Characteristic** | **Value (n = 44)** |
| --- | --- |
| Age at surgery, years (Mean ± SD) | 3.88 ± 2.15 (Range, 1–9) |
| Gender (Male / Female), n (%) | 28 (63.6%) / 16 (36.4%) |
| Affected Side (Right / Left), n (%) | 24 (54.5%) / 20 (45.5%) |
| Follow-up interval, months (Mean ± SD) | 49.3 ± 8.2 (Range, 30–68) |
| Failure of prior conservative therapy | 44 (100%) |

**Table 2. Summary of Perioperative Parameters and Functional Outcomes Following Endoscopic Sub-platysmal Release**

| **Parameter** | **Preoperative  Median (IQR)** | **Postoperative  Median (IQR)** | **P-value** |
| --- | --- | --- | --- |
| Rotation deficiency (degrees) | 26.5° (23.1° - 29.4°) | 3.0° (2.5° - 4.1°) | < 0.001 |
| Lateral bending deficiency (degrees) | 18.2° (15.8° - 20.5°) | 2.5° (2.0° - 3.3°) | < 0.001 |
| Operative time (minutes) | - | 35.2 ± 6.4 (Mean ± SD) | - |
| Intraoperative blood loss (mL) | - | 4.5 ± 1.2 (Mean ± SD) | - |
| Postoperative VSS score | - | 1.0 (0.0 - 1.0) |  |
| Complication rate (%) | - | 0 (0%) | - |

**Table 3. Longitudinal Follow-up of Range of Motion in Three Representative Cases**

| **Patient (Age)** | **Parameter (degrees)** | **Preoperative** | **Immediate Postoperative** | **12-Month Follow-up** |
| --- | --- | --- | --- | --- |
| Case 1 (3 years) | Rotation deficiency | 28 | 2 | 3 |
|  | Lateral bending deficiency | 20 | 1 | 2 |
| Case 2 (5 years) | Rotation deficiency | 24 | 3 | 3 |
|  | Lateral bending deficiency | 17 | 2 | 2 |
| Case 3 (8 years) | Rotation deficiency | 32 | 5 | 6 |
|  | Lateral bending deficiency | 22 | 4 | 4 |

**Table 4. Comparison of Postoperative Cheng and Tang Scores Across Age Groups**

| **Age Subgroup** | **N** | **Cheng and Tang Total Score (Mean ± SD)** | **P-value (Kruskal-Wallis)** |
| --- | --- | --- | --- |
| Toddler (1-2 years) | 14 | 17.6 ± 0.5 | 0.012* |
| Preschool (3-6 years) | 22 | 16.8 ± 1.2 |  |
| School-age (7-9 years) | 8 | 14.5 ± 2.0 |  |
